# Augmented reality during parotid surgery: real-life evaluation of voice control of a head mounted display

**DOI:** 10.1007/s00405-022-07699-8

**Published:** 2022-10-21

**Authors:** Claudia Scherl, David Männle, Nicole Rotter, Jürgen Hesser, Jan Stallkamp, Tobias Balkenhol, Lena Huber, Benedikt Kramer, Anne Lammert, Annette Affolter

**Affiliations:** 1 Department of Otorhinolaryngology, Head and Neck Surgery, University Medical Center Mannheim, Medical Faculty Mannheim, Heidelberg University, Theodor-Kutzer-Ufer 1-3, 68167 Mannheim, Germany; 2 Mannheim Institute for Intelligent Systems in Medicine, Medical Faculty Mannheim, Heidelberg University, Theodor Kutzer-Ufer 1-3, 68167 Mannheim, Germany

**Keywords:** Voice control, Augmented reality, HoloLens, Parotid surgery, Salivary glands, Head mounted display

## Abstract

**Purpose:**

Augmented Reality can improve surgical planning and performance in parotid surgery. For easier application we implemented a voice control manual for our augmented reality system. The aim of the study was to evaluate the feasibility of the voice control in real-life situations.

**Methods:**

We used the HoloLens 1^®^ (Microsoft Corporation) with a special speech recognition software for parotid surgery. The evaluation took place in a audiometry cubicle and during real surgical procedures. Voice commands were used to display various 3D structures of the patient with the HoloLens 1^®^. Commands had different variations (male/female, 65 dB SPL)/louder, various structures).

**Results:**

In silence, 100% of commands were recognized. If the volume of the operation room (OR) background noise exceeds 42 dB, the recognition rate decreases significantly, and it drops below 40% at > 60 dB SPL. With constant speech volume at 65 dB SPL male speakers had a significant better recognition rate than female speakers (p = 0.046). Higher speech volumes can compensate this effect. The recognition rate depends on the type of background noise. Mixed OR noise (52 dB(A)) reduced the detection rate significantly compared to single suction noise at 52 dB(A) (*p* ≤ 0.00001). The recognition rate was significantly better in the OR than in the audio cubicle (*p* = 0.00013 both genders, 0.0086 female, and 0.0036 male).

**Conclusions:**

The recognition rate of voice commands can be enhanced by increasing the speech volume and by singularizing ambient noises. The detection rate depends on the loudness of the OR noise. Male voices are understood significantly better than female voices.

## Introduction

The standard treatment for tumor lesions in the parotid gland is surgical removal. Since important nerves, especially the facial nerve, blood vessels, and muscles are located in this area, parotid surgery must be performed with particular caution. It is still difficult to guarantee sufficient surgical accuracy. In recent years, many surgical specialties have researched augmented reality (AR) to improve accuracy in surgical planning and performance: neurosurgery [1], urology [2], orthopedics [3], and general surgery [4], among others.

We have developed an AR system for a head mounted display (HMD) that facilitates orientation during parotid surgery [5]. In this system, all controls were performed using hand gestures. Although this has the advantage of contactless working conditions, it also conceals disadvantages: (i) surgical instruments have to be put down to carry out the gestures, (ii) workflow is interrupted, and (iii) frequent and long-lasting gestures are physically demanding. The system needs to be easy to use without prolonging the surgery or physically tiring the surgeon by intensive use of hand gestures. To simplify the way of working, we have developed a voice control for operating the HMD (HoloLens 1^®^). We have further developed the built-in commands, so that it can be applied more directly and individually to parotid surgery. The voice commands should be understood promptly during a surgery despite background noise. Therefore, our goal was to develop a measuring system to evaluate speech control. To our knowledge, there have been no systematic studies on speech control function of HMDs in the medical field yet. Our focus was to gain first trial experience concerning recognition rate depending on various background noises and other influencing factors, such as gender-specific voice pitch or command volume. Background noise plays an important role, especially when used in the OR. Aim of the study was to gain a first impression of the applicability of speech control and identify single factors that should be improved in the course of implementation of the devices in the surgical routine.

## Methods

### Augmented reality system

We used a head mounted display (Microsoft HoloLens 1^®^ (Microsoft Corporation, Redmond, USA)) as an AR device. The holographic module was developed in C# using Unity 3D (Unity 2018.4.22; Unity Technologies, San Francisco, USA) [6] and the Microsoft Mixed Reality Toolkit (MRTK Version 2.2; Microsoft Corporation, Redmond, USA). Exemplary patient data were extracted from DICOM MRI data and segmented using 3D Slicer 4.10.2 r28257 [7, 8]. The head models were rendered with a mean polygon count of 549,363 (SEM: 101,624, SD 287,435). Microsoft Visual Studio Community 2017 Version 15.9.23 [9] was used for software development and application deployment running on Microsoft Window 10 Education. For the creation of exemplary 3D vision of tumor and parotid gland we used the workflow described in our previous study [5]. Interactive manipulation of the holographic scene and objects was implemented using a hands-free approach (DFC-SYSTEMS GmbH, Munich, Germany) based on voice recognition features that has been developed specifically for our parotid surgery AR system described in [5, 10]. The study was approved by the Ethics Committee of the Medical Faculty Mannheim of Heidelberg University (Mannheim, Germany), (ethics vote no. 2019-739-AF5).

### Audiometry cubicle

**Fig. 1:**
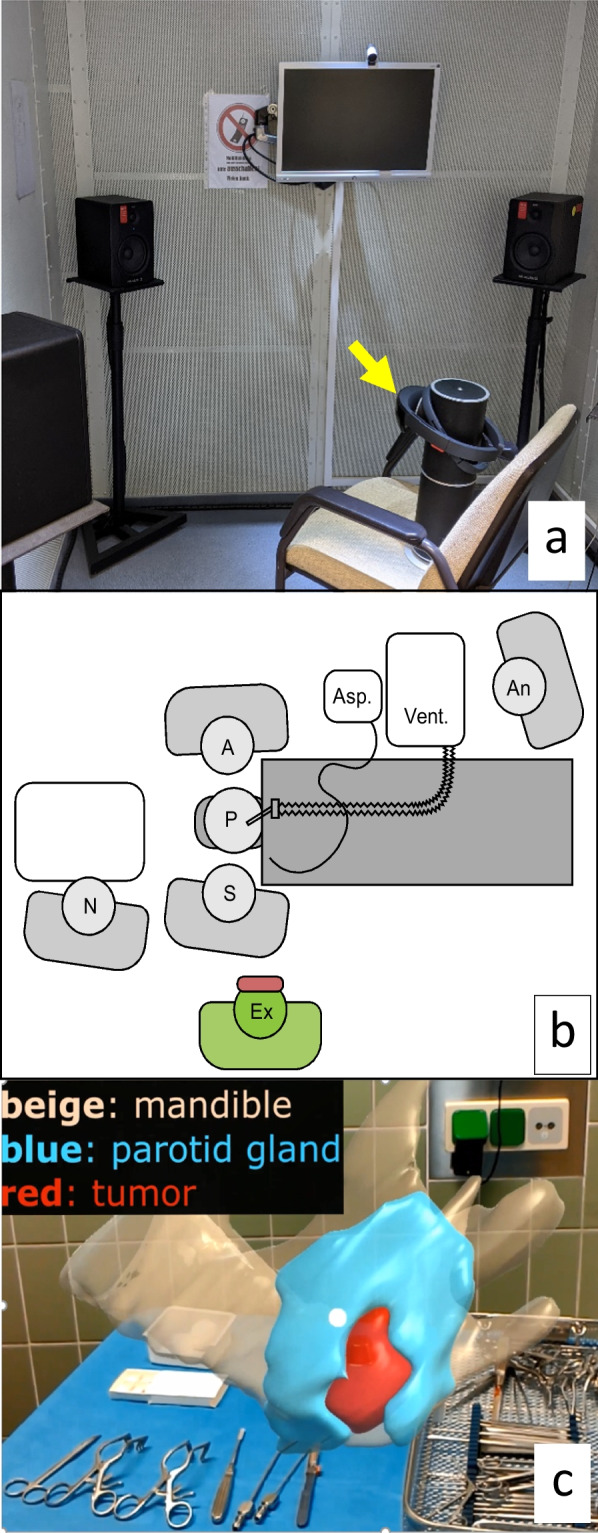
Measurement setup and holographic output. **a** Audiometry cubicle. Head mounted display, HoloLens 1^®^ (arrow) was placed centrally in IAC 403-A audiometry cubicle (IAC Acoustics, Niederkrüchten, Germany). The loudspeakers surrounded the device. The HMD was facing the front loudspeaker. M-Audio Fast Track Ultra USB Audio Interface and BX5 near-field monitor loudspeaker (inMusic Brand, Cumberland, RI, USA) were used for presenting the voice commands and background noise. **b** OR measurement setup. The experimenter (Ex) is wearing the HMD and stands closely behind the surgeon (S), who is next to the nurse (*N*) and across an assistant (A). The noise of the aspirator (Asp.) and the monitoring and ventilation unit (Vent.) is the main background noise. The laminar flow AC is another source of noise. The patient (P) is monitored by the anesthesiologist (An). Communication between the OR team is also considered as noise. Sound pressure measurements in audiometry cubicle and OR were performed with type 2250 sound level meter (Brüel & Kjær, Nærum, Denmark). **c** Example of holographic output showing a 3D view of the parotid gland, tumor and mandible. The views were altered by voice control: structures can be shown or hidden

To carry out reproducible and adaptable measurements, we carried out examinations in a DIN EN ISO 8253 certified audiometry cubicle. The cubicle enables to systematically measure various audiological parameters, such as type and sound pressure levels of the background noise, pitch of the speech commands, and volume to artificially adapt these parameters during measurement. Loudspeakers of the cubicle were used to play background noise (static noise, operation room noise). The type of the background noise also plays an important role for adequate command recognition. Static singular noise was compared to OR noise which is made up of a multitude of sounds. The noise of a suction system was chosen for the singular static noise. Measurement categories for different degrees of loudness were defined. Rendered 3D structures that are important for AR assisted parotid surgery as described in our previous work [5] have been assigned different voice commands. These recorded voice commands were played via an extra speaker next to the HMD (Fig. 1a). All voice commands were presented as male and female voices. All correctly recognized commands were registered.

### Operation room

In addition to the measurements in the audiometry cubicle, measurements were also done in the OR during ongoing surgeries (Fig. 1b) The median background noise in the OR was 56.9 dB(A) sound pressure level (SPL). Prerecorded voice commands were presented with a defined volume of 65 dB SPL as well as spoken commands with a volume intuitively adjusted to the OR noise. Static singular noise was compared to OR noise which is made up of a multitude of sounds. The noise of a suction system was chosen for the singular static noise.

### Statistics

Data were statistically analyzed using IBM SPSS Statistics for Windows, Version 20.0. Values were expressed as the mean ± standard deviation. Mean recognition rate of voice commands was compared between different background noises, gender, location (audio cubicle or OR) and commands using an unpaired sample t test. A *p* value < 0.05 was considered as significant for all tests.

## Results

Interactive manipulation of the holographic scene, such as hiding and showing of structures such as tumor lesions or the parotid gland (Fig. 1c) is controlled verbally. The quality and usability of this speech control under operative background noise was evaluated.

**Fig. 2:**
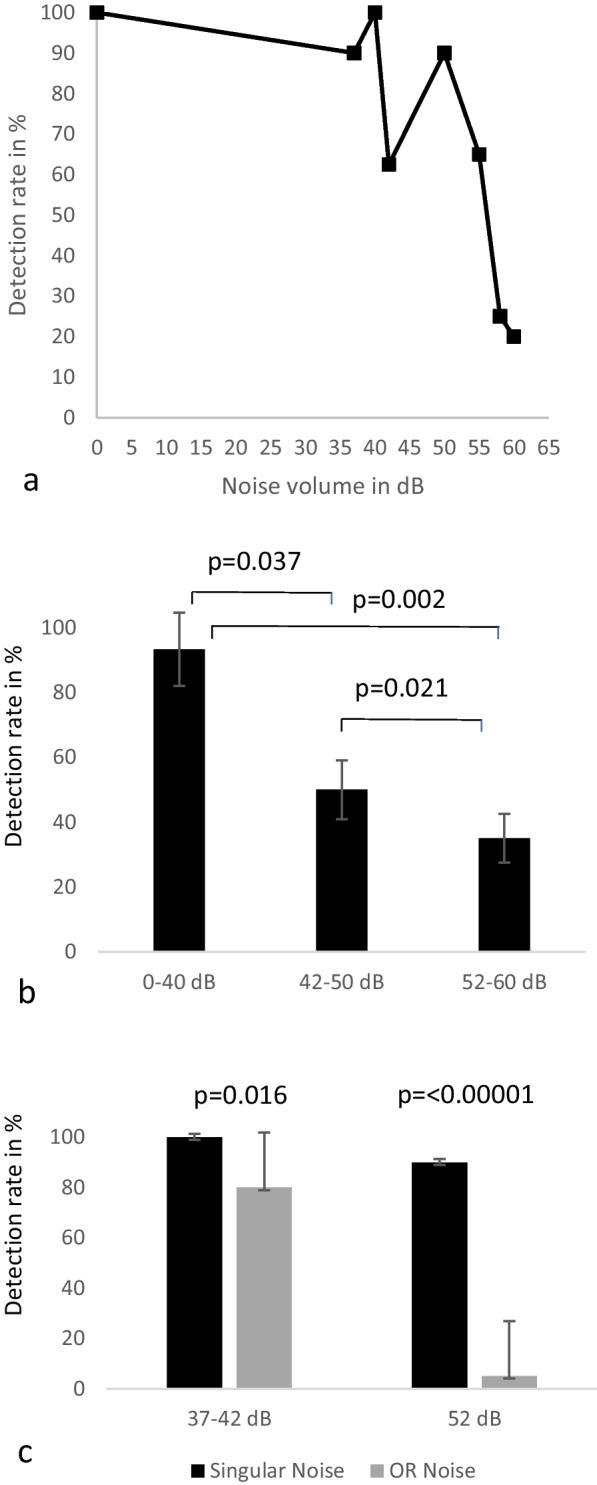
Detection rate in percent depending on the volume of the background noise (**a**, **b**) and on the type of noise (**c**). Normalized OR noise was used as background noise. Voice commands were presented at 65 dB SPL. **a** Detection rate decreases with increasing SPL. **b** Significant differences between individual volume groups. (*p* < 0.05 = significant). **c** Under normalized OR background noise, voice commands are registered significantly worse than under singular static background noise (e.g., suction). The louder the background noise, the greater the difference

Considering different levels of volume of prerecorded OR noise the recognition rate of the voice commands decreased with increasing SPL. To independently assess the recognition rate, voice commands were offered at a constant volume of 65 dB SPL. In silence 100% of commands were recognized. If the volume of the OR background noise exceeds 42 dB, the recognition rate decreased significantly (*p* = 0.002) and dropped below 40% (Fig. 2a, b).

We observed a significantly higher recognition rate (*p* = 0.016; < 0.00001) if there is only a static noise in the background when comparing the two background sounds, singular static and OR noise., This effect increased drastically with increasing volume (Fig. 2c).

Table 1 shows the difference of detection rates in the audiometry cubicle and measurements during real surgeries. The measurements took place at a noise level of 52 to 56.9 dB(A). The voice commands were given with a sound pressure level of 65 dB SPL or intuitively adjusted to the ambient noise level. Voices of different genders were considered. The recognition rate was significantly better in the OR than in the audio cubicle (*p* = 0.00013, 0.0086, and 0.0036).

**Table 1:** Recognition rates

Gender	Location	dB SPL	*N*	*N*	%	SD	*p* value
		commands	measures	identified			
m and f	AC	65	40	2	5		
m and f	OR	Intuitive	40	15	37.5	9.19	0.00013**
f	AC	65	20	1	5		
f	OR	Intuitive	20	7	35	4.24	0.0086**
m	AC	65	20	1	5		
m	OR	Intuitive	20	8	40	4.95	0.0036**
m	OR	Intuitive	20	8	40		
f	OR	Intuitive	20	7	35	0.71	0.376
m	AC	65	130	87	67		
f	AC	65	130	79	61	5.66	0.0460*

Difference of detection rates in the audiometry cubicle and measurements during real surgeries are shown. The table discriminates gender, location, and dB sound pressure levels of commands. In the OR commands were recognized significantly better than in the audio cubicle (*p* = 0.00013, 0.0086, and 0.0036)

*AC* audiometry cubicle, *m* male, *f* female, *N* number, *SD* standard deviation; *p* < 0.05* = significant; *p* < 0.01** = highly significant SPL of background noises 52-56.9 dB(A)

In the standardized conditions of the audio cubicle (volume of commands 65 dB SPL, background noise 52–56.9 dB(A)), male voice commands are recognized significantly better than female ones (*p* = 0.0460). At the measurements during surgery there was no significant difference between male and female voices (*p* = 0.376, Table 1) and in the recognition rate between different commands, such as “parotid” or “tumor”, respectively (*p* = 0.119).

## Discussion

The use of a HMD with voice control was first mentioned in 1998 for neurosurgery [11]. Many other surgical users also mention voice control [12–14]. So far, the usability under surgical requirements has not been examined in detail. This experimental study offers a proof of concept of the feasibility of a wearable AR device for using three-dimensional virtual object reconstructions controlled by voice commands. The tests took place in a surgical setting with disruptive factors occurring during every surgery. Speech control is a rapidly developing technology. Today, the majority of people are used to controlling PCs or smartphones via voice commands. The rapid evolution of low-cost user-to-information immersion interfaces such as voice-controlled HMDs have attracted significant attention from clinicians. Mojica et al*.* [15] mentions that voice commands for HMD control was praised and found highly desirable in visualizing MRI data.

Many publications report on gesture control for the HoloLens [15–17]. To date, no reports have been published that examine the technical applicability of using a voice control for HMD in a surgical setting. The ability to control the device with speech that does not require any physical movements would be ideal for surgical procedures in which interruption by putting down instruments for hand-gestures prolongs the surgery. However, this requires three conditions: (i) a stable voice control system, (ii) easy to pronounce commands, (iii) voice commands need to be recognized in a noisy environment, even when wearing a surgical mask.

Nguyen et al. [12] described an HMD controlled ultrasound system in which voice controls remove the need for the practitioners to interact physically with the ultrasound machine. During surgery, it is very important that the workflow is not interrupted by taking your eyes off the surgical site or by putting down the instruments. Both are necessary to execute gesture control. There is evidence that a disrupted visual-motor axis during surgery can lead to a plethora of problems including declined ergonomics and surgical performance, spatial disorientation, and increased risk of iatrogenic injuries [18]. Surgeons should maintain attention and focus on the patient. Our audiometrically supported study showed that speech control is possible and a potentially alternative control for AR devices to achieve more ergonomic and intuitive usability.

However, the built-in voice control system of the HoloLens is not yet optimally adapted to run in noisy environments. Across the globe, operating theatres are known to have high levels of noise, average noise levels range between 51 dB(A) and 79 dB(A) [19]. Median background noise during our OR measurements was 56.9 dB SPL. Noise in the OR is largely generated by machinery or environment moderating systems [20]. Our results show that command recognition rates decrease significantly with increasing mixed OR background noise levels. However, the device copes very well with singular background noise (e.g., suction). Even with very high SPLs (> 52 dB(A)), 90% of the commands are understood. This is considered a positive result, because noise from suction devices run continuously in the background in almost every surgery.

The recognition rate was better in the OR than in the audiometric cubicle, although the SPL of the background volume was the same. An essential factor for this phenomenon is the individual adjustment of the speech volume of the commander. The speech commands in the audiometry cubicle test setting were strictly given at 65 dB SPL. In the OR, this could not be done without problems. Therefore, the speech commands were spoken live by the study persons. They unknowingly adjusted the volume of the speech to the volume level in the OR resulting in a higher detection rate. This effect is well known. It has been determined that for an auditory signal to be understood with an accuracy of 90%, it has to be 10–15 dB HL louder than the background noise [21]. Thus, surgical staff must speak at a higher volume [22], in our case at least at a volume of 67–72 dB SPL.

The difference between women's and men's voices can be equalized in this way. Females and males have different voice pitches and subtle differences in syllable pronunciation. In our measurements, the command recognition rate differed significantly between male and female commands (*p* = 0.046). Commands spoken by men were understood significantly better regardless of the type of command itself. One reason for this lies in unequal training data. Male voices are primarily used for training automatic speech recognition systems and are, therefore, better recognized [23]. When women were able to unconsciously adjust command volume to noise volume, we found no gender differences anymore.

### Limitations

However, it must be mentioned that this analysis was considered a pilot project as little is known about speech control function of HMDs so far. Our intention was rather to describe than to improve the recognition rate which is a limitation of the current study, although it was not seen as to be in the frame of the analysis. Other limitations are due to the rather limited scope of a pilot study. It is difficult to accrue a high series of non-automated measures in a pilot project. We managed to run 20 to 130 test runs for each group. However, the homogeneous structure within these groups made it possible to achieve statistically significant values by comparison. Despite such limitations, data already indicate that recognition rate not only depends volume of commands and background voice but also on individual factors, such as gender characteristics. The recognition of voice commands in mixed background noise consisting of a multitude of sounds must be improved to implement voice control in daily surgical routines. Overall, this study utilized various audiological parameters with the goal of providing deeper insight to the yet elusive recognition rate of voice controlled HMDs in clinical use. However, the purposes of these devices have to be clearly and individually optimized, which will be addressed in future studies.

As shown by Navkar et al*.* [24] this study generally demonstrates that future development must be directed toward identifying an intuitive alternative to gesture control by voice commands with respect to 3D and 4D information.

## Conclusions

A special voice control system for a holographic AR interface for parotid surgery was implemented for interactive visualization of 3D anatomic structures. The recognition rate of the voice commands can be positively influenced by: (i) individual adjustment of the volume of the commands and (ii) by singularizing ambient noises.

The detection rate depends on the SPL of the OR noise. Male voices are understood significantly better than female voices.

## Data Availability

Not applicable.
